# Minimally Invasive Approach Utilizing Linear Stapler for Midline Incisional Hernia: Stapler Repair Technique

**DOI:** 10.1002/ags3.70026

**Published:** 2025-04-21

**Authors:** Sho Ueda, Takuya Saito, Kohei Yasui, Kentaro Shinohara, Yasuyuki Fukami, Kenitiro Kaneko, Tsuyoshi Sano

**Affiliations:** ^1^ Department of Gastroenterological Surgery Aichi Medical University Aichi Japan

**Keywords:** incisional hernia repair, intraperitoneal onlay mesh, laparoscopic Rives‐Stoppa approach, linear stapler, minimally invasive surgery

## Abstract

**Aim:**

We successfully established the stapler repair technique (SRT), a straightforward laparoscopic Rives‐Stoppa approach utilizing a linear stapler. This study retrospectively evaluated its short‐term outcomes to determine its safety and efficacy.

**Methods:**

The surgical outcomes of 87 patients who underwent laparoscopic median incisional hernia repair at our hospital were reviewed between August 2017 and May 2024. Patients were treated with intraperitoneal onlay mesh (IPOM), laparoscopic trans‐abdominal retromuscular (TARM), or SRT.

**Results:**

Among these patients, 37 were treated with IPOM, 16 with TARM, and 34 with SRT, with no significant differences in patient characteristics. The median surgical time (range) was 96 min (50–211) for IPOM, 256 min (196–300) for TARM, and 112 min (60–289) for SRT, respectively. The median mesh areas (ranges) were 210 cm^2^ (80–500) for IPOM, 500 cm^2^ (270–780) for TARM, and 379 cm^2^ (176–864) for SRT, respectively. The SRT group had significantly shorter operative times (*p* < 0.001) and smaller mesh areas (*p* = 0.005) than the TARM group. Compared to the IPOM group, there was no significant difference in operative time in the SRT group (*p* = 0.444), but the mesh area was significantly larger (*p* < 0.001). The SRT group had no significant intraoperative complications or conversions to open surgery.

**Conclusion:**

SRT offers a comparable operative time to IPOM and a significantly shorter time than TARM. Additionally, SRT can be performed extraperitoneally with no significant intraoperative complications or conversion to open surgery. These findings suggest that SRT is a safe and effective minimally invasive approach in median laparoscopic incisional hernia repair.

## Graphical abstract

Stapler‐assisted midline hernia repair.

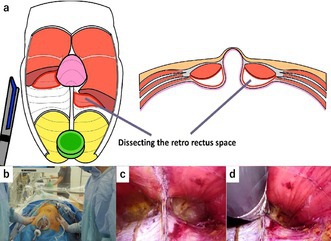

## 1 Introduction

Although the benefits of using a mesh for incisional hernia repair are well documented, there is still debate and disagreement regarding the optimal surgical technique and mesh placement [1, 2, 3, 4]. A lower recurrence rate has been reported with mesh implantation in the retro‐rectus space than inlay or onlay placement [2]. The laparoscopic approach has been shown to have a recurrence rate comparable to that of open repair, with superior outcomes in terms of length of stay (LOS) and surgical site infection (SSI) [5]. Intraperitoneal onlay mesh repair with hernia defect closure (IPOM) for laparoscopic incisional hernia repair is a common technique in many institutions [6, 7, 8, 9]. Recently, retro‐rectus mesh placement using laparoscopic methods has been described, such as the endoscopic mini/less open sublay technique (eMILOS) and the enhanced‐view total extraperitoneal technique (eTEP) [10, 11]. Nevertheless, the intricate process of dissecting the hernial sac and suturing the hernial orifice is time consuming and has not been widely accepted. Therefore, simpler techniques are required.

In 2016, Costa et al. published a laparoscopic Rives‐Stoppa technique called the Brazilian technique, which uses a linear stapler [12]. The procedure was performed on 15 patients with median incisional hernias after bariatric surgery and showed promising results. Based on this technique, we established a stapler repair technique (SRT).

This study aimed to evaluate the short‐term outcomes of laparoscopic median incisional hernia repair performed in our department and investigate SRT's safety and efficacy.

## 2 Methods

### 2.1 Patients

We retrospectively collected data from consecutive patients who underwent median laparoscopic incisional hernia repair at the Aichi Medical University between August 2017 and May 2024. All patients underwent abdominal and pelvic computed tomography (CT) for preoperative hernia measurement and operative planning. The inclusion criterion was a midline anterior abdominal wall incisional hernia between the xiphoid and the symphysis pubis. Hernias were measured using European Hernia Society (EHS) criteria [13]. The exclusion criteria included pregnant women, patients with cancer, and patients with clinical contraindications to the proposed method.

The protocol for this research project was approved by the ethics review committee of the Aichi Medical University (approval number #2023‐008). This study conformed to the provisions of the Declaration of Helsinki. Informed consent was obtained from all participants.

### 2.2 Surgical Technique for SRT

**FIGURE 1:**
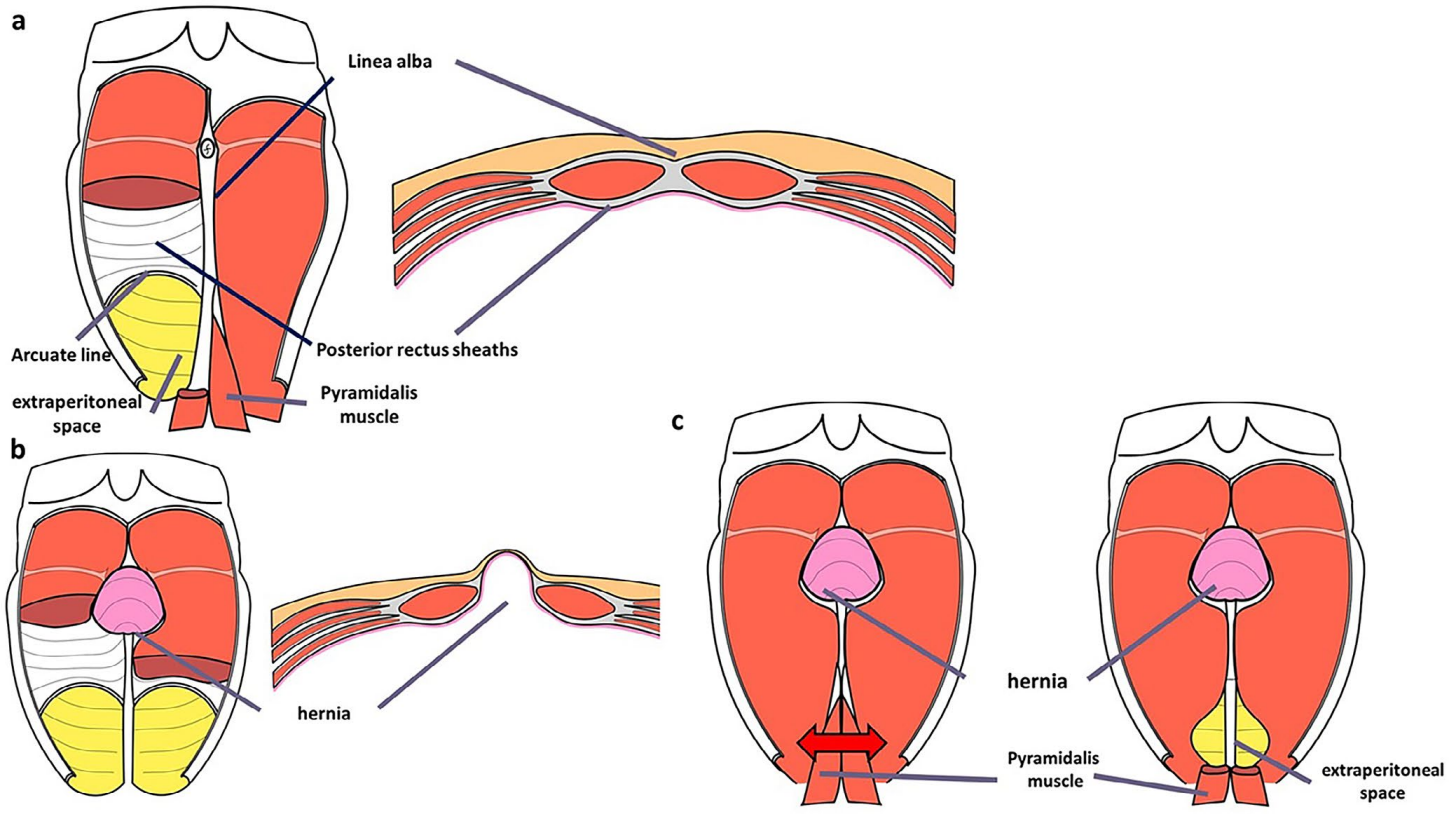
(a) A detailed schematic diagram of the abdominal wall anatomy. The top of the figure corresponds to the patient's head, while the bottom corresponds to the feet. (b) A schematic diagram illustrating a midline incisional hernia in the umbilical region. (c) Make a 4‐cm transverse incision in the midline of the lower abdomen, approximately two transverse finger widths above the pubic symphysis. The anterior rectus sheath and the pyramidal muscle are dissected to access the extraperitoneal space within the retrorectus space.

**FIGURE 2:**
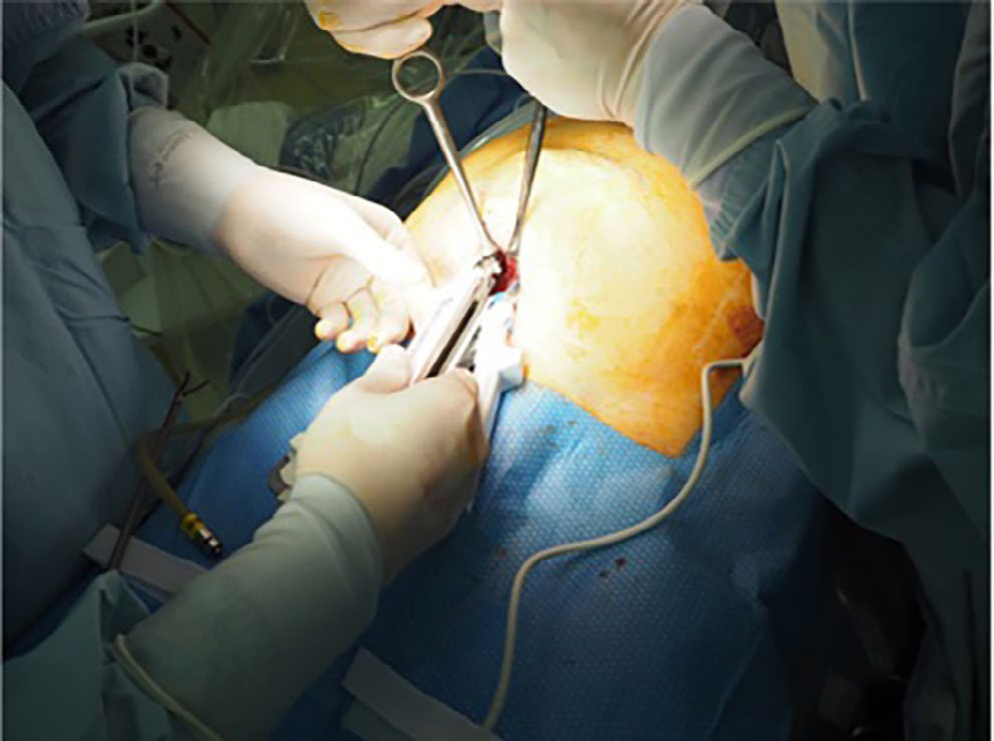
After the left and right retro‐rectus spaces are detached toward the head, a 75‐mm linear stapler is inserted, and the linea alba is stapled.

**FIGURE 3:**
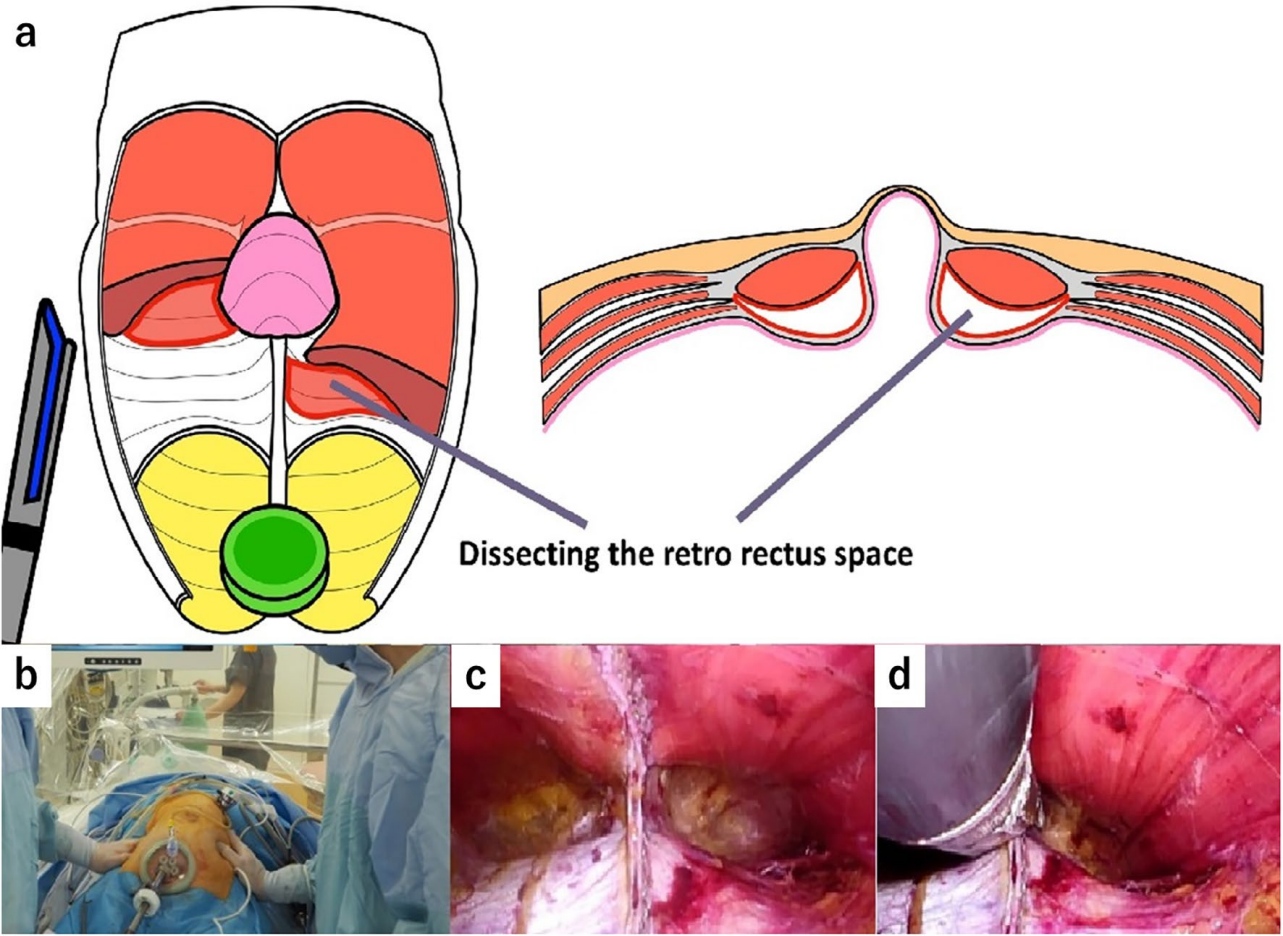
(a) The retractor is placed, and both retro‐rectus spaces are dissected toward the head in a laparoscopic maneuver. (b) Insert Echelon black 60 mm (ETHICON) through a 12‐mm port placed in the retractor. (c) Both retro‐rectus spaces are dissected and ready for the second stapling. (d) Stapling the linea alba and hernia sac toward the head side.

**FIGURE 4:**
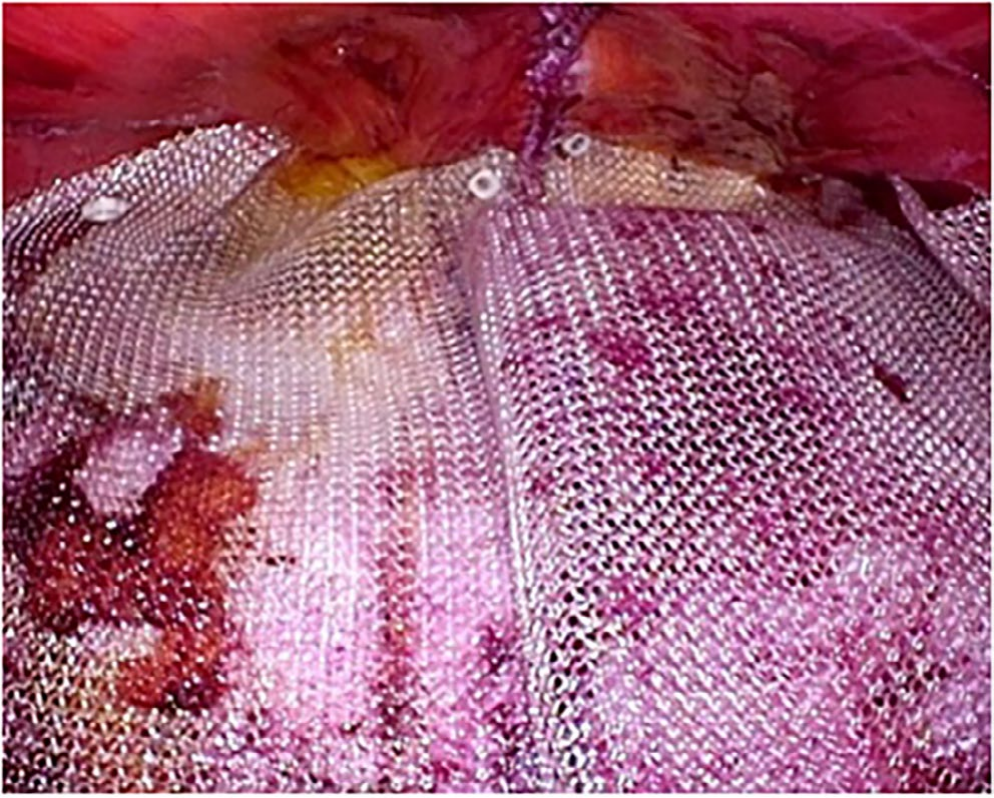
The mesh should be trimmed to the dissection distance plus 3 cm on either side, keeping a margin of 5 cm cephalocaudally from the hernia portal. The left and right sides are designed to be slightly wider and rolled ventrally. Fix the mesh with a tacker at 5–6 points.

In the original Brazilian technique, stapling is performed via an intraperitoneal maneuver, whereas in the SRT, it is performed via a completely extraperitoneal route. The procedure is as follows:

1. Patient positioning: After initiation of general anesthesia, position the patient supine with hands out and hips flexed dorsally by 10°–15° to avoid obstruction of forceps manipulation. The operator stands to the right of the patient.
2. Port placement: Using the optical technique, insert a 5‐mm port at Palmer's point, 3 cm below the left rib arch. Observe the abdominal cavity. If adhesions are present, add a port on the left side of the abdomen, as needed.
3. Incision and space creation: Make a 4‐cm transverse incision in the midline of the lower abdomen, approximately two transverse finger widths above the pubic symphysis. Extend the incision through the anterior rectus sheath, entering the retro‐rectus space. Simultaneously, the pyramidalis muscle is separated. If there is a surgical scar, identify and dissect the pyramidalis muscle to enter the extraperitoneal space (Figure 1a–c). Dissect the dorsal side of the rectus muscle to avoid the formation of an arteriovenous vein in the inferior abdominal wall.
4. Stapler insertion: Secure the extraperitoneal space caudal to the arcuate line. Manually dissect the retro‐rectus space toward the head. After 7 cm of the linea alba dissection on both sides, insert a 75‐mm linear stapler caudally toward the head (Figure 2). Align the stapler slowly and medially, compressing the lateral abdominal areas and the staple while loosening the tension. Check for intestinal pinching before stapling, which is mandatory.
  The first stapling is performed under direct vision. Due to the proximity of the stapler and camera in laparoscopic operation, interference between the stapler and camera prevents proper stapling. The assistant elevates the abdominal wall to expand the visual field, allowing the surgeon to insert the 75‐mm linear stapler and perform stapling under direct vision. However, as the tip of the stapler is not visible, the procedure remains somewhat blind. To overcome this limitation, although limited to cases where the hernia orifice is located cranially to the umbilicus (EHS classification M1, M2, M3), a recent approach involves laparoscopically dissecting the linea alba up to the umbilical region before performing all stapling under laparoscopic guidance. Video S1 provides a detailed visual demonstration of this stapling process and laparoscopic assistance, further illustrating key technical aspects of the procedure.
5. Port and retractor placement: Place the Smart Retractor S size (top) in the retro‐rectus space, apply a cap, and insert a 5‐mm and a 12‐mm port. Adjust the cap so the 5‐mm port is slightly to the left of the patient's head. Insert a 5‐mm flexible camera to develop the field of view. Perform blunt dissection with Solacotton or a sharp incision with the ultrasonic coagulation cutting device through the 12‐mm port toward the head, especially on both sides of the linea alba (Figure 3a). Insert an Echelon black 60‐mm (ETHICON) ventrally and perform a second stapling (Figure 3b–d). This stapling from this position improves the interference between the camera and the stapler, allowing stapling to be performed under laparoscopic observation.
6. Further dissection and stapling: The visual field should be easier to secure after the third stapling. Dissect the right and left sides until the neurovascular bundle (NVB) entering the posterior rectus sheath is visible. Close and transect the hernia portal along with the linea alba using stapling. Continue stapling toward the head until 5 cm from the hernia portal is secured. Check for intestinal pinching within the abdominal cavity each time.
7. Mesh placement: Use polypropylene mesh. Maintain a 5 cm margin from the hernia orifice to the cephalocaudal side, trimming the left and right sides to the detached distance plus 3 cm. Fix the mesh in place at five or six points using a tacker (Figure 4).
8. Drain placement: Reinsert the left subseptal port into the extraperitoneal space and place a drain through this port.

However, in cases classified as EHS M5, where the hernia orifice is located suprapubically, a supraumbilical approach is required instead of a suprapubic one. Once the retro‐rectus space is accessed, the procedure follows the same steps, with the direction of the dissection and stapling adjusted cranially or caudally, depending on the hernia location.

### 2.3 Statistical Analysis

Our institution developed SRT in 2022. We compared the surgical outcomes of SRT with the IPOM and laparoscopic trans‐abdominal retromuscular (TARM) methods, which were the preferred techniques before the introduction of SRT. Continuous data were expressed as median (range). Statistical analyses were performed using the chi‐squared test, Mann–Whitney *U*‐test, Fisher's exact probability test, Kruskal–Wallis test, and Steel test, as appropriate. Statistical significance was set at *p* < 0.05. All statistical analyses were performed using EZR for R software (R Project for Statistical Computing, Vienna, Austria) [14].

## 3 Results

### 3.1 Patient Characteristics

Eighty‐seven patients underwent median laparoscopic incisional hernia repair at our hospital. Among them, 37, 16, and 34 were treated with IPOM, TARM, and SRT, respectively. The clinical characteristics of the patients are summarized in Table 1. No significant differences were observed in clinical characteristics between the groups.

**TABLE 1:** Patient characteristics.

	IPOM (*n* = 37)	TARM (*n* = 16)	SRT (*n* = 34)	*p*
Age (year)	73 (32–90)	71 (47–87)	72 (43–87)	0.962
Sex (male/female)	14/23	4/12	15/19	0.545
Height (cm)	158 (140–175)	153 (140–175)	159 (143–172)	0.562
Body weight (kg)	62 (39–89)	60 (49–86)	64 (40–112)	0.743
BMI	26.4 (16.0–32.4)	26.1 (17.2–36.7)	25.1 (18.7–39.7)	0.845
Hernia orifice (cm^2^)	25.0 (4.0–198.0)	22.5 (2.0–224.0)	23.5 (4.0–80.0)	0.418
EHS classification				
M1/M2/M3/M4/M5	0/8/20/6/3	0/0/14/2/0	0/6/24/2/2	0.188

*Note:* Expressed as median (range).

### 3.2 Perioperative Outcomes

Table 2 shows the patients' perioperative variables. The outcomes of SRT were compared with those of IPOM and TARM.

**TABLE 2:** Perioperative outcomes.

	IPOM (*n* = 37)	TARM (*n* = 16)	SRT (*n* = 34)	*p*	*p*
				IPOM vs. SRT	TARM vs. SRT
Mesh area (cm^2^)	210 (80–500)	500 (270–780)	379 (176–864)	< 0.001	0.005
Operation time (min)	96 (50–211)	257 (196–300)	112 (60–289)	0.444	< 0.001
Blood loss (ml)	2 (0–320)	4 (0–30)	9 (0–114)	0.047	0.375
Days in hospital (day)	6 (2–28)	6 (4–15)	8 (4–22)	0.119	0.942
Complication (*n*)	1 (2.7%)	3 (19%)	0 (0%)	0.04	
	Bleeding	SSIx2, Bleeding			
Laparotomy transition (*n*)	3 (8.1%)	0 (0%)	0 (0%)	0.07	
Reoccurrence (*n*)	2 (5.4%)	0 (0%)	0 (0%)	0.144	

*Note:* Expressed as *N* (%) or median (range).

**FIGURE 5:**
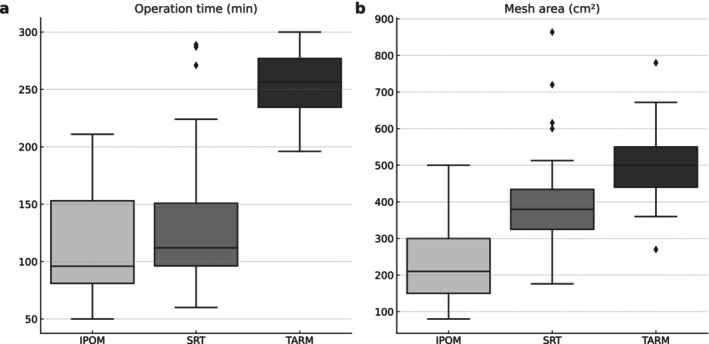
(a) SRT had a significantly shorter operative time compared with TARM (*p* < 0.001) and was not substantially different from IPOM (*p* = 0.444). (b) SRT had a significantly smaller mesh area compared with TARM (*p* = 0.005) and a significantly larger mesh area compared with IPOM (*p* < 0.001).

The operating times were 96 min (range, 50–211 min) for IPOM, 256 min (range, 196–300 min) for TARM, and 112 min (range, 60–289 min) for SRT. SRT had a significantly shorter operative time than TARM (*p* < 0.001) and was not substantially different from IPOM (*p* = 0.444) (Figure 5a).

The mesh areas were 210 cm^2^ (range, 80–500 cm^2^) for IPOM, 500 cm^2^ (range, 270–780 cm^2^) for TARM, and 379 cm^2^ (range, 176–864 cm^2^) for SRT. SRT had a significantly smaller mesh area than TARM (*p* = 0.005) and a significantly larger mesh area than IPOM (*p* < 0.001) (Figure 5b).

In terms of blood loss, 9 mL (range, 0–114 mL) of blood loss for SRT was significantly different from the 2 mL (range, 0–320 mL) for IPOM (*p* = 0.047), but not from the 4 mL (range, 0–30 mL) for TARM (*p* = 0.375). There were no significant differences in the LOS between the groups.

Postoperative complications included one case of postoperative bleeding in the IPOM group, which did not require a blood transfusion, two cases of surgical site infection, and one case of postoperative bleeding in the TARM group, also not requiring transfusion. No complications were observed in the SRT group. All complications had a Clavien–Dindo classification [15] of II or lower.

Three patients were converted to laparotomy in the IPOM group, and no patients were converted to laparotomy in the TARM or SRT groups. However, two patients in the SRT group were converted to IPOM due to stapling failure (Table 3). The stapling failure in both cases was due to excessive tension at the hernia defect, which caused the stapler to break and fail to close the defect (unstable stapler closure). In particular, Case No. 1 had a history of multiple laparotomies, leading to significant scar formation at the hernia defect, which further increased tension during closure. Notably, stapling failure was observed only in M3 cases, while no failures were reported in M5 cases, which required a supraumbilical approach.

**TABLE 3:** Details of cases converted from SRT to IPOM due to stapling failure.

Case No.	Age	Sex	BMI	Comorbidities	EHS classification	Hernia size (cm)	Stapling failure reason	Stapler firings attempted	Conversion criteria	Operative time (min)	Blood loss (mL)	Hospital stay (days)	Recurrence (follow‐up)
1	72	M	19.8	Multiple laparotomies	M3	8 × 6	Excessive tension	1	Unstable stapler closure	287	207	8	No (12 months)
2	61	F	27.4	None	M3	7 × 7	Excessive tension	1	Unstable stapler closure	140	0	10	No (6 months)

Two patients in the IPOM group experienced recurrence, whereas no recurrences were reported in the TARM or SRT groups. The median follow‐up period was 3.5 years (range, 0.5–7 years) for IPOM, 3 years (range, 2–3 years) for TARM, and 1 year (range, 0.5–2 years) for SRT.

## 4 Discussion

In this study, we evaluated the short‐term outcomes of our SRT method for median laparoscopic incisional hernia repair. We compared these results with those of the IPOM and TARM methods.

Our results demonstrate that SRT offers a significantly shorter operative time than TARM and a comparable operative time to IPOM. This suggests that SRT may be a more efficient option, particularly compared with the more time‐consuming TARM method. Additionally, SRT uses a significantly smaller mesh area than TARM does which could indicate a less invasive approach while maintaining adequate coverage and support for hernia repair. Conversely, the mesh area for SRT was larger than that for IPOM, possibly reflecting extraperitoneal mesh placement, which requires more extensive dissection and coverage.

There were no significant differences in hospital stay between the groups, suggesting that SRT is comparable with respect to this perioperative outcome. However, there was a slight but significant difference in blood loss between the IPOM and SRT groups, with the SRT group showing slightly higher blood loss. Despite this, blood loss in all groups remained relatively low and was considered clinically harmless. Notably, the SRT group experienced no postoperative complications, whereas the IPOM and TARM groups experienced postoperative bleeding and surgical site infections. This may indicate a potential advantage of SRT in terms of postoperative safety.

The conversion rates to laparotomy differed notably between the groups. The IPOM group had three cases of conversion, while the SRT group had none, although two SRT cases were converted to IPOM due to stapling failure. As shown in Table 3, both cases of stapling failure involved excessive tension at the hernia defect, which caused the stapler to break open upon the first firing, preventing closure. This suggests that the success of the first stapling is critical in determining the overall success of the SRT technique. If excessive tension is observed when clamping the hernia defect with a linear stapler, component separation (CS) or other tension‐reducing techniques should be considered before attempting closure.

We have added an inguinal single‐port approach of endoscopic component separation (ECS) in cases with a large hernia orifice [16]. The ECS technique was added in two cases where stapling failed, indicating that stapling can be particularly challenging in cases with large transverse widths of the hernia orifice. This highlights a potential challenge of the stapling technique in SRT, which may require further refinement to reduce the need for conversion. To reduce the incidence of stapling failures, there is an interest in preoperative treatments that may assist in reducing the size of hernia defects. Although not covered by insurance in Japan, botulinum toxin treatment has shown promise [17, 18].

Two patients in the IPOM group experienced recurrence, whereas no recurrence was reported in the TARM and SRT groups during the follow‐up period. This suggests that SRT may provide durable repair with a low recurrence rate comparable to, or potentially better than, that of IPOM. However, since SRT is a relatively new technique, the follow‐up period remains shorter, which is a limitation in assessing its long‐term durability.

One of the significant advantages of SRT is that it allows for the simultaneous division and closure of the hernia defect using a stapler. This ensures secure closure of the hernia defect and reinforces both the anterior and posterior walls of the abdominal wall.

An advantage of SRT is that it does not require technically demanding laparoscopic suturing or a crossover maneuver that involves cutting the linea alba and connecting the left and right extraperitoneal spaces at the midline. However, a drawback of SRT is the high costs associated with stapler use.

However, while the high costs associated with stapler use are a drawback of the SRT method, this disadvantage may be offset by the reduced operative time, lower incidence of complications, and the relative simplicity of the procedure.

Additionally, implementing a tacker‐less approach could reduce costs, enhancing the procedure's economic efficiency.

Our findings suggested that SRT is a promising laparoscopic median incisional hernia repair technique. It offers an efficient operative time, adequate mesh coverage, and favorable perioperative outcomes, with minimal complications and low recurrence rates. Further studies with larger sample sizes and more extended follow‐up periods are needed to confirm these results and fully establish the safety and efficacy of SRT.

## Author Contributions

**Sho Ueda:** data curation, formal analysis, investigation, writing – original draft. **Takuya Saito:** methodology, project administration, supervision, writing – review and editing. **Kohei Yasui:** visualization, writing – review and editing. **Kentaro Shinohara:** writing – review and editing. **Yasuyuki Fukami:** writing – review and editing. **Kenitiro Kaneko:** writing – review and editing. **Tsuyoshi Sano:** project administration, supervision, writing – review and editing.

## Ethics Statement

This study was approved by the Ethics Review Committee of Aichi Medical University (approval number #2023‐008) and conformed to the provisions of the Declaration of Helsinki. Informed consent was obtained from all participants.

## Conflicts of Interest

The authors declare no conflicts of interest.

## Supporting information


**Video S1.** A step‐by‐step demonstration of the stapler repair technique (SRT) for midline incisional hernia repair. The video highlights key surgical steps, including port placement, retro‐rectus space creation, stapling technique, and mesh placement under laparoscopic guidance.
