# Correction to “Minimally Invasive Approach Utilizing Linear Stapler for Midline Incisional Hernia: Stapler Repair Technique”

**DOI:** 10.1002/ags3.70192

**Published:** 2026-02-15

S. Ueda, T. Saito, K. Yasui, K. Shinohara, Y. Fukami, K. Kaneko, and T. Sano, “Minimally Invasive Approach Utilizing Linear Stapler for Midline Incisional Hernia: Stapler Repair Technique,” *Annals of Gastroenterological Surgery* 9, no. 5 (2025): 1086–1092. https://doi.org/10.1002/ags3.70026.

In the **Methods, Section 2.3 | Statistical Analysis**, the following sentence is incorrect:

“Our institution developed SRT in 2022. We compared the surgical outcomes of SRT with those of IPOM and laparoscopic trans‐abdominal retromuscular (TARM) methods, which were the preferred techniques before the introduction of SRT.”

It should read as:

“Our institution developed SRT in 2022. We compared the surgical outcomes of SRT with those of IPOM and laparoscopic trans‐abdominal retromuscular (TARM) methods*, which were the preferred techniques before the introduction of SRT.”

The following reference should also be included as follows:

*Masurkar AA. “Laparoscopic Trans‐Abdominal Retromuscular (TARM) Repair for Ventral Hernia: A Novel, Low‐Cost Technique for Sublay and Posterior Component Separation,” *World J Surg*. 2020;44(4):1081–1085. https://doi.org/10.1007/s00268‐019‐05298‐z.

We apologize for this error.

